# Factors associated with dynamic balance in people with Persistent Postural Perceptual Dizziness (PPPD): a cross-sectional study using a virtual-reality Four Square Step Test

**DOI:** 10.1186/s12984-021-00852-0

**Published:** 2021-03-25

**Authors:** Moshe M. H. Aharoni, Anat V. Lubetzky, Liraz Arie, Tal Krasovsky

**Affiliations:** 1 Department of Physical Therapy, Faculty of Social Welfare and Health Sciences, University of Haifa, Haifa, Israel; 2 Department of Physical Therapy, Steinhardt School of Culture Education and Human Development, New York University, New York, NY USA; 3 Pediatric Rehabilitation Department, Sheba Medical Center, Ramat Gan, Israel

**Keywords:** FSST, Anxiety, Kinematics, HTC Vive, Visual stimuli, Chronic dizziness

## Abstract

**Background:**

Persistent postural-perceptual dizziness (PPPD) is a condition characterized by chronic subjective dizziness and exacerbated by visual stimuli or upright movement. Typical balance tests do not replicate the environments known to increase symptoms in people with PPPD—crowded places with moving objects. Using a virtual reality system, we quantified dynamic balance in people with PPPD and healthy controls in diverse visual conditions.

**Methods:**

Twenty-two individuals with PPPD and 29 controls performed a square-shaped fast walking task (Four-Square Step Test Virtual Reality—FSST-VR) using a head-mounted-display (HTC Vive) under 3 visual conditions (empty train platform; people moving; people and trains moving). Head kinematics was used to measure task duration, movement smoothness and anterior–posterior (AP) and medio-lateral (ML) ranges of movement (ROM). Heart rate (HR) was monitored using a chest-band. Participants also completed a functional mobility test (Timed-Up-and-Go; TUG) and questionnaires measuring anxiety (State-Trait Anxiety Inventory; STAI), balance confidence (Activities-Specific Balance Confidence; ABC), perceived disability (Dizziness Handicap Inventory) and simulator sickness (Simulator Sickness Questionnaire). Main effects of visual load and group and associations between performance, functional and self-reported outcomes were examined.

**Results:**

State anxiety and simulator sickness did not increase following testing. AP-ROM and HR increased with high visual load in both groups (p < 0.05). There were no significant between-group differences in head kinematics. In the high visual load conditions, high trait anxiety and longer TUG duration were moderately associated with reduced AP and ML-ROM in the PPPD group and low ABC and  high perceived disability were associated with reduced AP-ROM (|r| =  0.47 to 0.53; p < 0.05). In contrast, in controls high STAI-trait, low ABC and longer TUG duration were associated with increased AP-ROM (|r| = 0.38 to 0.46; p < 0.05) and longer TUG duration was associated with increased ML-ROM (r = 0.53, p < 0.01).

**Conclusions:**

FSST-VR may shed light on movement strategies in PPPD beyond task duration. While no main effect of group was observed, the distinct associations with self-reported and functional outcomes, identified using spatial head kinematics, suggest that some people with PPPD reduce head degrees of freedom when performing a dynamic balance task. This supports a potential link between spatial perception and PPPD symptomatology.

## Introduction

Persistent-postural perceptual dizziness (PPPD) is a condition characterized by chronic vestibular symptoms [1]. These symptoms may include subjective dizziness, instability or both, which persist over 3 months. PPPD is the most common vestibular diagnosis, estimated at 15%–20% of people with complaints of vestibular symptoms [2], and is associated with increased levels of anxiety [3]. People with PPPD often seek recognition for their complaints [4], yet, with physical and laboratory tests typically remaining normal, the mechanism underlying the symptomatology of this condition remains unclear. A recent study showed that people with PPPD demonstrate impaired spatial navigation capabilities when performing a navigation task while sitting and manipulating a joystick in virtual reality [5]. Spatial disorientation is known to be related to balance disorders [6] yet its implications to functional complaints and balance performance in individuals with PPPD is unknown.

Individuals with PPPD typically experience exacerbation of their symptoms during upright self-motion [2, 3, 7] and/or during exposure to complex full-field visual stimuli [2]. The mechanism of this is thought to be related to poor sensory integration in individuals with PPPD, namely increased reliance on vision for balance [2, 8, 9]. Increased visual reliance in PPPD has been shown to be associated with balance performance [11, 12] and likelihood to develop chronic symptoms [13]. In addition, in studies evaluating postural control in conditions which share features with the newly-defined PPPD, such as visual vertigo [14] or phobic postural vertigo [15], moving visual stimuli or deprivation of visual input generated increased postural sway, altered head and trunk kinematics or reduced gait speed. These findings suggest that assessing balance impairments and spatial disorientation in individuals with PPPD needs to be done in the presence of complex full-field visual stimuli. Nevertheless, typical balance tests do not replicate the complex visual environments encountered in everyday life. Furthermore, functional mobility [16] and psychological traits like anxiety [17] are known to relate to dynamic balance performance in different populations. Anxiety can interfere with visuomotor control of gait, and it has been shown that anxious older adults rely more on vision for individual step control [18]. It is possible that in people with PPPD, factors such as anxiety may further impair dynamic balance performance. Indeed, in a recent model of visual control of posture, Bronstein [10] demonstrated how both reliance on vision and psychological variables (e.g. anxiety) can affect postural motor responses by altering the central processes which effectively mediate visual-vestibular sensory weighting.

**Fig. 1:**
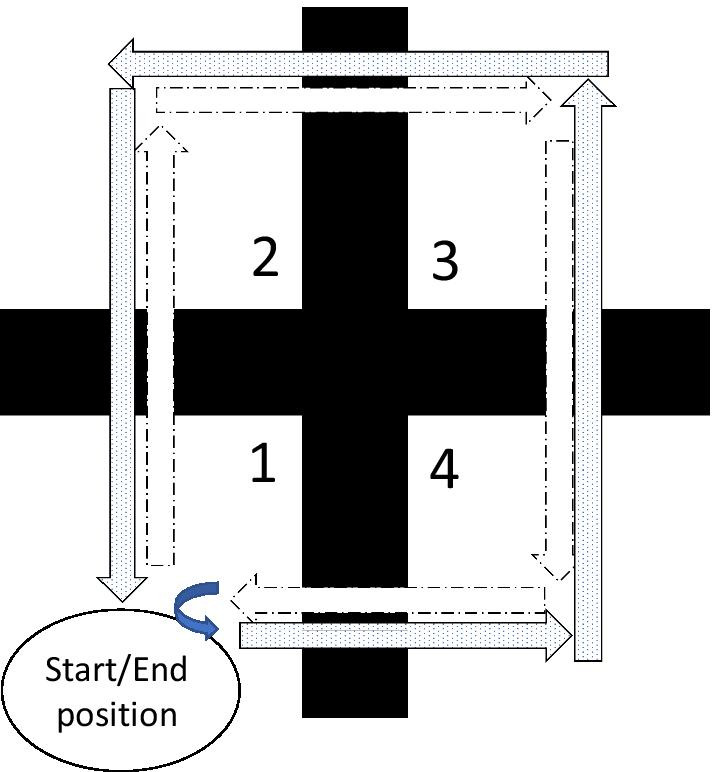
The traditional four-square step test sequence: a participant is asked to walk as fast as possible clockwise (broken arrows) and counterclockwise (dotted arrows) over four walking canes arranged in a cross-shaped form, while looking forward. The participant should step through using both feet, as the starting and ending point are similar (square 1)

The four-square step test (FSST) [19, 20] is a test of dynamic balance originally developed for older adults. The FSST measures time for completion of a rapid sequential square-shaped stepping task clockwise and counterclockwise while avoiding stepping canes (or marks [21]) placed on the ground (Fig. 1).

The rapid stepping task requires changes in direction, and challenges motor planning and coordination more than straight-line walking. Over the years, the FSST has been validated as a functional test of balance for various clinical populations such as people with Parkinson's disease, stroke or vestibular disorders [22]. In people with vestibular disorders, a cutoff of 12 s or longer suggests increased fall risk [19]. The FSST is conducted in a well-lit room with no visual distractions. In addition, because the outcome of the FSST is duration of performance in seconds, the clinical FSST does not provide any information on movement strategy and quality of performance beyond speed. In this work, we transferred the existing FSST [19, 20] into a virtual environment (FSST-VR). Virtual reality (VR) is increasingly used for studying balance in individuals with dizziness and balance impairments [23–26]. In the current study, we used a head-mounted display (HMD) that projects semi-realistic immersive visual experiences (HTC Vive; [27]) and was shown to be a valid and reliable tool for measuring postural tasks [28]. We chose to develop the FSST-VR to quantify dynamic balance for several reasons. First, it allowed us to compare performance of people with PPPD and controls on the FSST under different conditions of salient, mild visual load, such that they may encounter in their daily living. In addition to the ability to simulate a rich environment with moving visual stimuli which may be experienced in everyday life, the FSST-VR requires participants to move within this complex environment. Finally, HMDs accurately track head kinematics and can therefore go beyond duration of performance to shed light on movement strategy and spatial orientation during movement [29, 30]. Taken together, findings regarding head kinematics in complex salient visual environments could have major clinical implications, due to the portability, ease of use and cost of the HMD setup.

### Objectives

The current study goal was to quantify dynamic balance performance in individuals with PPPD under various levels of visual load. Specifically, the aims of the current study were: (1) to evaluate the feasibility of using the FSST-VR in individuals with PPPD and healthy individuals, both in terms of potential adverse events such as increased state anxiety or cybersickness and in comparing performance duration with the traditional FSST (2) to assess spatiotemporal kinematic parameters of FSST-VR in people with PPPD compared to healthy individuals under different visual load conditions, and (3) to evaluate the association between self-reported measures, functional mobility tests and dynamic balance performance of people with PPPD and controls during the FSST-VR.

## Methods

This work is a part of a multi-site research project conducted at New York University and University of Haifa. Data collection occurred in both sites. Preliminary results from this work were previously presented [31].

### Participants

Participants with PPPD over the age of 18 were recruited via physicians, physical therapists, university advertising and social networks, and frequency-matched for age and gender with healthy participants. Participants in the PPPD group were diagnosed according to the ICD-11/Bárány Society diagnostic criteria [2] as confirmed via a phone interview. Participants reported feelings of movement, dizziness, unsteadiness or light-headedness over a period of > 3 months, exacerbated by movement and/or busy visual environments. Previously conducted examinations included bloodwork, cardiac testing, brain and inner ear imaging and additional negative tests of hearing, vision, touch and vestibular function (for details see [32]). Exclusion criteria for both groups were: a history of drug/alcohol abuse; active neuro-otologic disorders other than PPPD; new medication use or recent change in dosage less than one month prior to participation; pregnancy; neurological conditions affecting balance; musculoskeletal pain affecting gait or standing; impaired cognition; peripheral neuropathy and uncorrected visual impairments.

### Procedure

**Fig. 2:**
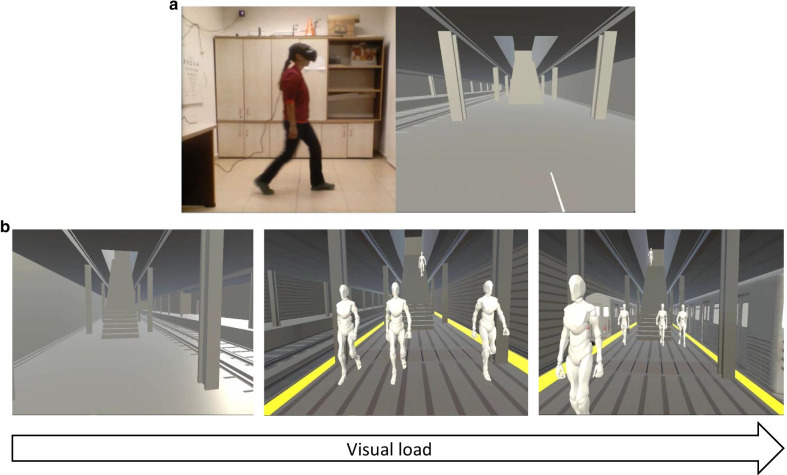
FSST-VR visual conditions: **a** Left: An individual wearing the head mounted display while performing the FSST-VR. Right: the respective virtual environment viewed via the HMD. **b** Varying visual loads, from simple (left): empty subway platform, to complex (center): people moving in the AP direction; to Complex + (right): additional trains moving around the platform

A detailed description of the procedure was previously published [31]. Participants were asked to fill questionnaires regarding state and trait anxiety (State-Trait Anxiety Inventory; STAI [33]), perceived disability due to dizziness (Dizziness Handicap Inventory, DHI [34]) and balance confidence (Activities-specific Balance Confidence scale; ABC [35]) and perform functional mobility tests including the Timed-Up and Go test (TUG [36]) and the traditional FSST using its original instructions [20]. Participants were asked to perform the FSST within the virtual environment (VE, a subway platform), under one of 3 visual conditions (Fig. 2): Simple (empty platform), Complex (people moving in anteroposterior direction), Complex + (same as complex with trains passing by). The FSST-VR did not include physical canes, but rather a virtual cross drawn on the floor in the VE [21]. Participants were verbally instructed as follows: "As in the FSST performed earlier, try and complete the sequence as fast as possible, stepping over the virtual lines and looking straight ahead". Looking down during the trial (lowering the head) resulted in repeating the trial.

The FSST-VR task was repeated 3 times for each condition. The Simple condition was always introduced first—a 60–120 s practice trial followed by a recording of 3 repetitions of the FSST-VR, and the Complex conditions followed in a randomized order. Participants wore on their chests a heart rate monitor (H10, Polar, Finland) synchronized with a mobile phone. The Simulator Sickness Questionnaire (SSQ) and the STAI-state were completed prior to the FSST-VR and immediately afterwards. This task was part of larger VR protocol where all participants performed the FSST-VR at the end. SSQ values reported here reflect scores immediately before and after the FSST-VR.

Head position was recorded using the HTC Vive and spatiotemporal head kinematics were extracted using custom-written MatLab code (version R2018a, Mathworks, Natick, MA). The head’s tangential velocity profile was calculated filtered using a dual-pass low-pass Butterworth filter (20 Hz cutoff). Performance duration (time to complete FSST-VR) was calculated based on onset and offset times determined automatically as the first and last moment when the tangential velocity profile exceeded 10% of peak velocity. In addition, as a control measure, an investigator timed the task duration using a stopwatch. Smoothness of movement was defined by the number of peaks in the tangential velocity profile per repetition. Since more peaks represent a jerkier movement, for maximal smoothness the FSST-VR should consist of 8 peaks—one for each step. Spatial outcomes from the FSST-VR included AP and ML ranges of movement of the head (AP-ROM, ML-ROM). Heart rate was extracted from the mobile phone app and averaged for each trial.

### Statistical analysis

Sample size was determined a-priori. In order to detect a small effect size (d = 0.25) with α = 0.05 and 95% power (for a within-between interaction repeated-measures analysis of variance), a total of 54 people was required. Normality of all demographic and study variables were tested using Kolmogorov–Smirnov test and the choice of tests was done accordingly. Changes in state anxiety and simulator sickness pre-post testing were evaluated using Wilcoxon signed-rank test. Performance duration differences between the FSST-VR and the traditional FSST and between manual and automatic timing within and between groups were evaluated using paired and unpaired sample *t*-tests, and agreement between them was measured using intraclass correlation coefficients (ICC(2,1), single measures). The main effects of group and visual load were examined using a Repeated Measures Analysis of Variance for normally distributed variables, and Friedman’s test, Mann–Whitney U and Wilcoxon signed-rank tests for non-normally distributed variables. Spearman's rank correlation coefficient was used for determining the association between self-reported measures, functional mobility tests and FSST-VR performance outcomes. All statistics were calculated using SPSS (version 20, IBM, Armonk, NY).

## Results

### General

Fifty-two people participated, among them 29 controls and 23 people with PPPD. One participant was excluded from the PPPD group following outlier analysis. HR data for one control participant was missing in one condition due to technical reasons. Participant characteristics are described in Table 1. Participants reported mean symptom duration of 5.6 ± 7.8 years (range 0.4 to 30). In 16 participants, the symptoms appeared suddenly and in 2 participants the symptoms appeared gradually. Precipitating events to symptom onset were varied, and included stress (N = 5), panic attack (N = 3), a flu (N = 3), an episode of BPPV (N = 2), a bumpy flight/bus ride (N = 3), giving birth (N = 2) and an episode of alcohol consumption (N = 1). Event was unknown in 4 participants. People with PPPD had lower functional mobility (t_(49)_ = − 3.77, p < 0.01), lower balance confidence (Z = − 5.72, p < 0.01), more disability due to dizziness (Z = − 6.72, p < 0.01) and more trait anxiety (t_(49)_ = − 5.61, p < 0.01) than controls.

**Table 1:** Demographic, self-reported measures and functional testing scores

Outcomes	Group	
	*Controls* *(n* = *29)*	*PPPD* *(n* = *22)*
Demographic measures		
Gender (male/female)	11/18	12/10
Age (years)	30 [15.3]	32 [12.8]
Height (cm)	170 [13]	170.5 [18]
Weight (kg)	69.57 (14.86)	69.90 (14.58)
Self-reported measures		
STAI-trait (20–80)	35.83 (7.48)	51.36 (12.20)**
ABC (0–100)	98.13 [4.99]	76.3 [26.24]**
DHI (0–100)	0	54.60 (18.5)**
Functional testing scores		
TUG (s)	7.27 (1.43)	8.79 (1.40)**
FSST (s)	5.64 (1.23)	6.27 (1.21)

Values in the table are mean and (standard deviation) for normally distributed variables, and median and [interquartile range] for non-normally distributed variables.

*STAI-trait* Trait score on the State-Trait Anxiety Inventory, *ABC* Activities-specific Balance Confidence scale, *DHI/HDHI*  Dizziness Handicap Inventory/Hebrew Dizziness Handicap Inventory, *TUG* Timed-Up and Go test, *FSST*  Four Square Step Test. Statistically significant differences between groups are noted with ** (p < 0.01)

*Feasibility of FSST-VR:* No participants had any adverse effects during or following exposure to the virtual environment. As shown in Table 2, state anxiety (STAI-state) and simulator sickness (SSQ) levels immediately after exposure to the FSST-VR did not increase significantly in either group.

**Table 2:** Anxiety and simulator sickness levels before and after FSST-VR

	*Controls (n* = *29)*			*PPPD (n* = *22)*		
	Before	After	Median difference	Before	After	Median difference
STAI state score (20–80)	30 [12]	32 [12]	1 [9.25]	41 [14]	41.5 [15]	0.5 [7.25]
SSQ score (0–48)	2 [6]	2 [5]	0 [2.5]	8 [8]	12 [6]	1 [8.25]

Values in the table are median and [Interquartile Range]. STAI state = the State-Trait Anxiety Inventory—state; SSQ = Simulator Sickness Questionnaire

In both groups, longer durations were demonstrated for the FSST-VR in the simple environment compared with the traditional FSST with no VR (PPPD: t_(21)_ = 21.03, p < 0.01; Controls: t_(28)_ = 29.18, p < 0.01; Tables 1 and 3) with no between-group differences. Agreement between FSST-VR and traditional FSST was r = 0.69 (PPPD: r = 0.84, 95% CI 0.66–0.93; Controls: r = 0.53, 95% CI 0.20–0.75). When comparing the traditional FSST duration with the manual timing of the FSST-VR, no differences were noted between VR and non-VR versions, and agreement between them was r = 0.84 (PPPD: r = 0.9, 95% CI 0.77–0.96; Controls: r = 0.79, 95% CI 0.60–0.90) such that this difference can be attributed to the onset/offset detection algorithm’s sensitivity.

**Table 3:** Head kinematics and HR during performance of FSST-VR

Kinematic outcomes	Simple environment		Complex environment		Complex + environment	
	*Controls* *(n* = *29)*	*PPPD* *(n* = *22)*	*Controls* *(n* = *29)*	*PPPD* *(n* = *22)*	*Controls* *(n* = *29)*	*PPPD* *(n* = *22)*
Duration (s)	7.48 [7.10]	7.46 [2.06]	7.15 [1.25]	7.45 [1.55]	7.23 [1.74]	7.39 [1.68]
Number of peaks (N)	10.66 [2.75]	11 [3.42]	10.33 [2.33]	11 [1.25]	10.33 [2.33]	10.83 [2.58]
ML-ROM (m)	0.28 [0.08]	0.30 [0.06]	0.28 [0.05]	0.31 [0.08]	0.29 [0.08]	0.31 [0.05]
AP-ROM (m)	0.34 (0.07)	0.36 (0.06)	0.37 (0.09)	0.37 (0.79)	0.37 (0.09)	0.37 (0.79)
HR (bpm)	92.15 (17.93)	97.46 (10.93)	95.68 (18.77)	100.94 (11.64)	96.79 (20.10)	101.71 (11.34)

Values in the table are mean and (standard deviation) for normally-distributed variables, and median and [interquartile range] for non-normally-distributed variables. ML-ROM = Mediolateral range of motion; AP-ROM = Anteroposterior range of motion, *HR* heart rate

### Main effect of visual load and group

**Fig. 3:**
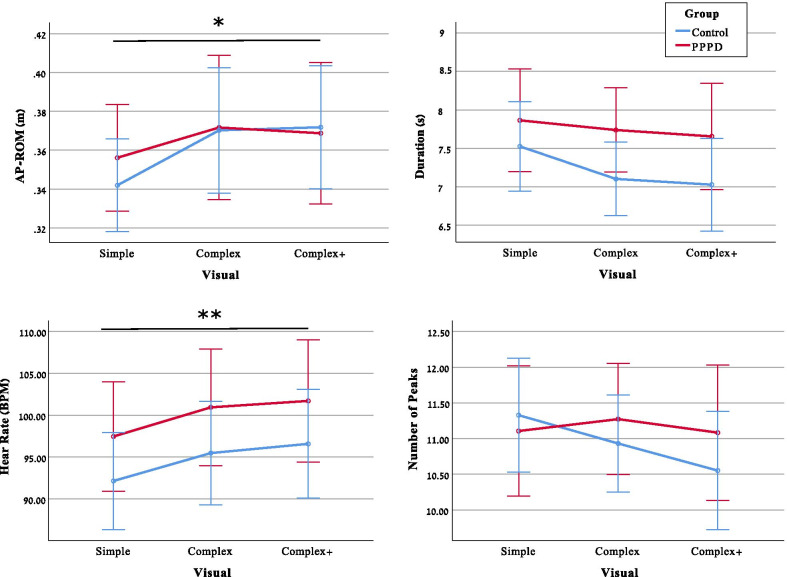
Results of Analysis of Variance (ANOVA) for kinematic variables and heart-rate across groups (red = PPPD, blue = Control) and conditions (Simple = empty platform, Complex = busy platform, and Complex +  = busy platform with trains). Error bars denote 95% confidence intervals

Kinematic parameters of FSST-VR in different conditions of visual load are listed in Table 3 and results of the ANOVAa are depicted in Fig. 3. AP-ROM increased with visual load in both groups (F_(2,98)_ = 3.77, p = 0.03, PES = 0.07). Visual load was also associated with increased HR, with no difference between groups (F_(2,96)_ = 13.37, p < 0.001, PES = 0.33). However, duration, smoothness or ML-ROM did not change with visual load in either group. There were no between-group differences in head kinematics or HR in all visual environments. Given the lack of differences between the more complex visual conditions, for all subsequent analyses conditions of visual load were pooled into low (simple) and high (average of Complex and Complex +).

### Association of self-reported measures and functional mobility tests with performance of FSST-VR

**Fig. 4:**
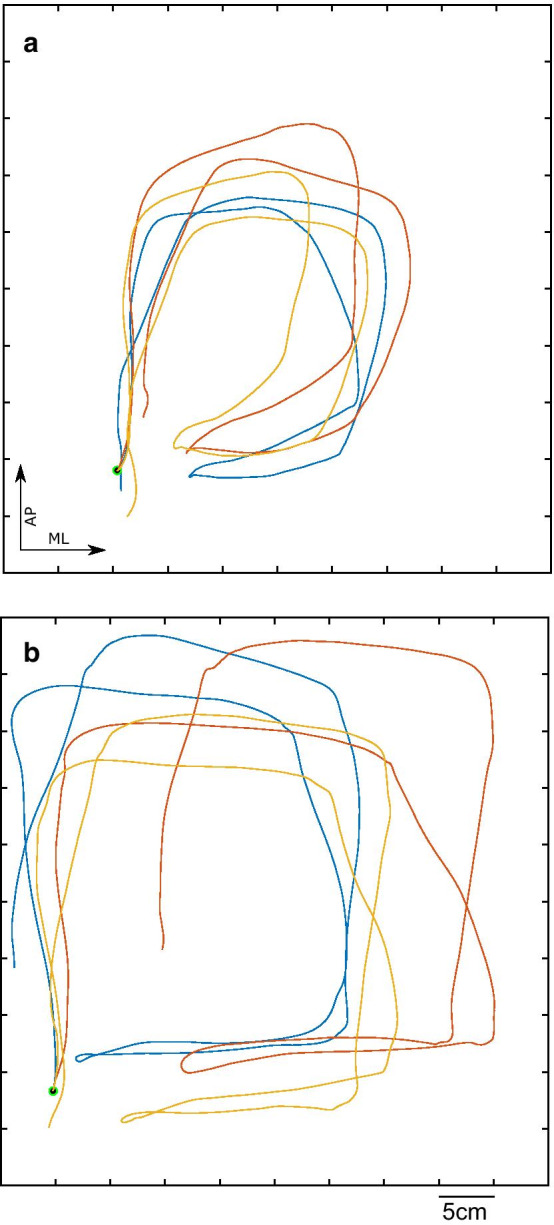
Three representative traces of head movement for two people with PPPD during performance of FSST-VR. Traces are aligned to a common starting point, marked by a black dot. **a** P02: STAI trait = 50, DHI = 84, ABC = 62.5. **b** P07: STAI trait = 25, DHI = 18, ABC = 88.8

**Fig. 5:**
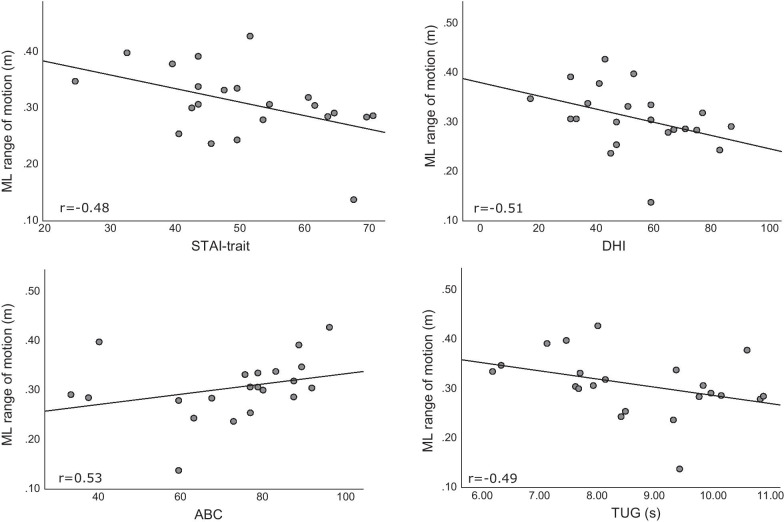
Correlations between self-reported and functional outcomes and mediolateral range of motion (m) in people with PPPD during performance of the FSST-VR under high visual load

An example of spatial trajectories for 2 representative participants with PPPD is provided in Fig. 4. The pattern demonstrated in panel A is of decreased ROM in both AP and ML directions for a participant with high trait anxiety, high perceived disability and low balance confidence. When examining the relationships between spatiotemporal variables and self-reported and functional mobility outcomes in both groups, two distinct patterns emerged. In the PPPD group self-reported factors such as high trait anxiety (STAI-trait), reduced balance confidence (ABC), and an increased sense of disability due to dizziness (DHI) were associated with reduced ML-ROM (Fig. 5). A similar association emerged for functional mobility, where reduced functional mobility (high TUG score) was associated with reduced ML-ROM; trait anxiety and functional mobility were associated with reduced AP-ROM as well (Table 4). These relationships were specific to the high visual load conditions. In contrast, among controls increased anxiety, reduced balance confidence and reduced functional mobility, were associated with an *increase* in AP-ROM. Functional mobility was further associated with increased ML-ROM in controls. These relationships were, again, specific to the high visual load conditions. HR was only positively associated with trait anxiety in controls, and increased movement smoothness was associated with better balance confidence and functional mobility in controls. Importantly, no relationships were identified between any factor and movement duration in either group.

**Table 4:** Associations of self-reported and functional outcomes with FSST-VR performance

Self-reported/functional outcomes	Spatiotemporal head kinematic outcomes				
	Number of peaks low (N)	AP ROM low (m)	AP ROM high (m)	ML ROM high (m)	HR low (bpm)
PPPD					
STAI-trait	− 0.08	− 0.11	− 0.47*	− 0.48*	0.04
ABC	− 0.18	0.26	0.37	0.53*	− 0.34
TUG	0.39	− 0.21	− 0.52*	− 0.49*	0.01
DHI	− 0.03	− 0.29	− 0.31	− 0.51*	0.25
Controls					
STAI-trait	− 0.2	0.34	0.38*	0.32	0.4*
ABC	0.45**	− 0.29	− 0.4*	− 0.22	− 0.18
TUG	− 0.50**	0.39*	0.46*	0.53**	0.02

Values in the table are Spearman's rank correlation coefficients and significance. * = p < 0.05, ** = 0 < 0.01**.**

Low = Simple visual environment; High = pooled Complex and Complex + visual environments; AP ROM = Anteroposterior forward–backward stepping; ML ROM = Mediolateral side-stepping; HR = heart rate; STAI-trait = the State-Trait Anxiety Inventory—trait; ABC = Activities-specific Balance Confidence scale; DHI = Dizziness Handicap Inventory/Hebrew Dizziness Handicap Inventory; TUG = Timed-Up and Go test; TMT-B = Trail making Test part B FSST = Four Square Step Test. Outcomes with no significant relationship with all variables were excluded from the table. These are, for both groups: Movement duration (low and high) and Number of peaks (high)

## Discussion

This work aimed to establish a virtual-reality balance assessment for people with PPPD. To date, no tailored VE was designed for balance assessment or intervention in this population [37]. Given the prevalence of this condition and its impact on quality of life, this is an important first step in the design of such environments. Our paradigm, of which preliminary results were published [31], was found to be feasible. First, we did not observe major changes in state anxiety, simulator sickness or heart rate. Second, between-group comparison of stopwatch performance and automatic measurement of movement duration from the headset showed similar results. Note that the automatic detection of movement onset and offset generated a constant bias in terms of movement duration. This suggests that head kinematics may be used in order to detect movement duration of FSST within virtual environments, but these 2 types of measurements (stopwatch and head kinematics) should not be directly compared. Performance duration of FSST-VR, like the traditional overground FSST, was similar between groups. While we did not identify between-group differences in performance of the FSST-VR task, we found moderate associations between self-reported and functional mobility outcomes with head kinematics during performance of the FSST-VR that were opposite between people with PPPD and controls specifically at the ‘high’ visual load environments.

The VEs in this study were designed to evaluate dynamic balance performance in individuals with PPPD under conditions of visual load similar to those which may be encountered in daily life. It should be noted that exacerbating symptoms was not the objective of the current work. Symptoms of people with PPPD may be exacerbated by continued, rather than one-time exposure to visual stimuli [2]. Thus, it is possible that repeating the protocol over an extended period would have caused more symptoms in people with PPPD. We devised 3 levels of visual load which were meant to simulate real-life conditions by modifying AP visual movement within the VE (avatars and/or trains) and the task included self-motion. However, we found no main effect of group on any spatiotemporal variable in the FSST-VR, and the main effect of visual load was similar between groups—in both groups AP-ROM increased with visual load. In addition, no significant differences were found in movement duration between the visual conditions. In the current study, as in that of others [7, 3] participants with PPPD had higher trait anxiety, increased disability due to dizziness, lower functional mobility and lower balance confidence compared with controls (Table 1). However, we did not observe between-group differences in FSST performance either overground or in VR. This may be because of the nature of balance performance in PPPD, which seems to lie along the spectrum of healthy variability in motor performance within the general population. Indeed, a recent study found symptoms of PPPD to be more prevalent in the general population than previously thought (with 50% of healthy participants scoring above the 25^th^ percentile when examined using a self-reported measure of visual symptoms associated with PPPD [38]). The current work extends these findings to suggest that people with PPPD may not differ from controls when dynamic balance performance is examined. This similarity suggests that people with PPPD may have effectively compensated for their limitations when performing this complex balance task. This compensation may be easier when the task requirement is to perform the movement as fast as possible, as was the case here. Indeed, previous work suggests that people with phobic postural vertigo, a precursor to PPPD, walk slower than controls when asked to walk at slow and at comfortable speeds, but at similar speed to controls when asked to walk fast [15]. These authors concluded that when requested to move fast, gait control may be shifted to a more automatic mode, requiring less integration of visual-vestibular input and enabling improved performance. This may be the case also for people with PPPD in the current work. It is possible that the requirement to walk fast in the FSST (overground or in VR) underlies the absence of between group differences regardless of the visual input. It is also possible that people with PPPD were able to direct attention away from the visual distractors due to the complexity of the motor task. Future work should examine variants of this task with different levels of moving visual stimuli and at different movement speeds. With and without an additional concurrent cognitive task [39] These future directions are relevant for the design of VEs for the assessment of balance control in people with PPPD.

Despite the lack of between-group differences, this study demonstrated a consistent moderate association between self-reported and functional mobility outcomes and head kinematics during performance of the FSST-VR, that was different between people with PPPD and controls. Specifically, within the PPPD group increased trait anxiety, perceived disability, reduced balance confidence and reduced functional mobility were associated with smaller range of motion in AP and ML directions. These relationships may suggest that people with PPPD who are more anxious and less confident limit movement in the VR, i.e. adopt a “high risk” stiffening movement strategy [17]. In contrast, among controls increased anxiety, decreased balance confidence and reduced functional mobility were associated with the opposite tendency—that of increased range of motion within the VE. These inverse relationships may underlie the variability and lack of between-group differences in the current study. Still, the FSST-VR and specifically the spatial measure of head ROM, were able to discern subtle alterations in task performance within both groups. These relationships suggest that potentially, a more fine-grained assessment of movement characteristics in people with PPPD, i.e. differentiation according to self-reported and functional characteristics, is required in order to reveal the origin of between-subject variability in symptomatology and performance of dynamic balance tasks. A recent study [5] suggests that a possible mechanism for PPPD symptomatology is an impairment in spatial navigation in the absence of reliable visual cues. Brain imaging studies identified decreased activity [40] and functional connectivity [41] XXXof the precuneus, an area associated with spatial processing, in people with PPPD. Results from the current study, which examined self-motion under complex visual load conditions (but with limited visual cues for self-motion), suggest that specific characteristics of PPPD are associated with limited ROM. Thus, the spatial aspect, rather than the temporal aspect of performance of this dynamic balance task, may be affected by PPPD.

The relationships identified between head kinematics, self-reported measures and functional mobility outcomes were specific to the high conditions of visual load. Previous work has shown that when performing a static balance task people with phobic postural vertigo (a precursor to PPPD as recently defined) demonstrate increased postural sway when the postural task is easier, but not when postural demands increase [42]. The increased postural demand may result in increased effort to stabilize. Our finding that people with PPPD who were more symptomatic moved less only in the high load conditions, suggests that the increased visual load, although not significantly disturbing performance for people in the PPPD group, may have been difficult enough to expose the role of specific self-reported and functional characteristics in people with PPPD. This finding needs to be examined in light of Bronstein’s model of visuo-postural control of balance [10]. According to this model, the effect of individual characteristics such as trait anxiety on postural control may be navigated via their impact on upregulation of visual cues (in lieu of vestibular and proprioceptive cues). This may explain the appearance of these relationships specifically in the high load visual conditions in the current work.

A particularly interesting relationship identified in our sample of people with PPPD is the negative relationship between trait anxiety and spatial ROM during the FSST-VR in high visual load. The FSST-VR is a complex balance task, requiring increased awareness of body location and orientation, as well as planning complex directional changes. It has been shown that when facing an anxiety-inducing postural threat, high attention to self-movement predicts subjective perception of instability in healthy older individuals under normal visual conditions [43]. It may be that people with PPPD and high trait anxiety, when faced with high visual loads, are worried of losing their balance, therefore freeze degrees of freedom and as a result limit spatial ROM during the task. This could be attributed to interoceptive avoidance [44] which is also the basis for a cognitive-behavioral model explaining PPPD [45]. Alternatively, it may be that people with PPPD and anxiety limit head range of motion only in order to stabilize the head in a top-to-bottom frame of reference [46]. Since kinematics of the feet was not measured in the current work, we are unable to discern between these possibilities. In any case, this strategy of limitation of movement paradoxically simplified the transitions required for efficient temporal performance of the task, as demonstrated in this study. A possible mechanism which can explain our finding of reduced self-motion in busy environments among people with PPPD and high trait anxiety was demonstrated in a recent study by Passamonti et al. [47]. In their study, functional brain connectivity was evaluated under conditions of very high visual load (virtual rollercoaster ride). Their work demonstrated that in people with PPPD, neuroticism was associated with more activation in the inferior frontal gyrus, and higher connectivity between this area and occipital regions [46]. These relationships, which were not identified in controls, may translate to increased attention to visual motion stimuli in busy environments and to limited self-motion, as demonstrated here. Further work is required, however, to evaluate the cognitive and sensory mechanisms underlying self-motion of people with PPPD within virtual environments.

The FSST, which was adapted here to a virtual environment, was originally designed to evaluate fall risk in older adults [20] and was validated for use with various clinical populations [22] including people with vestibular disorders [19]. In this work, we added outcome measures of spatiotemporal head kinematics, specifically head ROM in different movement directions, using a relatively inexpensive HMD (HTC Vive) which we have recently demonstrated to provide valid and reliable information of head kinematics in postural tasks [28]. Indeed, head movement ROM but not movement time were associated with functional characteristics in both groups in this study. This provided important information on dynamic balance which was missing from the overground version of the test. In recent years, a proliferation of use of wearable sensors is demonstrated in various fields of rehabilitation [48]. Our results highlight the importance of expanding the scope of kinematic outcomes from clinical tests when integrating them with novel technology [49, 50], as well as the feasibility of merging virtual reality applications with advanced motion sensing technology [48].

Some limitations to this study should be acknowledged. First, following the instructions of the overground FSST, the VR task instructions did not involve any reference to range of motion (only to performance speed and gaze). Indeed, decreased head ROM could have resulted in faster movement—but this was not the case here as movement time did not vary with personal characteristics, either self-reported or measured, in either group. An additional limitation involves the measure of ROM derived from head kinematics, which may not accurately represent stepping kinematics when markers are attached to the feet. Additional work will examine the relationships between head movement and stepping parameters during performance of the FSST-VR, specifically among people with PPPD, in order to ascertain the origin of decreased head ROM in the more severely affected participants. It is important to note that this work may have been underpowered to detect correlations between functional and kinematic characteristics for each group separately. While this will need to be replicated in future work with larger samples, note that the relationships identified were consistent in directions for the different measures within each group. The cross-sectional design of this work precluded the ability to determine causal relationships between the different self-reported and functional mobility outcomes and head kinematics. Indeed, while highly anxious individuals are more prone to develop PPPD [13], the fact that multiple factors were associated with FSST-VR performance raises the question of whether it is a condition-specific association or rather an effect associated with anxiety per se, as it is known that people with anxiety disorders have impairments in postural control [51]. Finally, the protocol was not fully randomized, rather the simplest condition was always introduced first. This was done due to our concern that due to the challenge in the FSST task, introducing it in VR could be overwhelming and provoking, particularly for people with PPPD. We wished to minimize these adverse effects and maximize patients’ comfort, and therefore opted out of full randomization and chose to introduce the simplest condition first. The current feasibility results indicate that in future studies, full randomization may be possible.

## Conclusions

The current work demonstrates that performance of an FSST task in VR is feasible for people with PPPD. Overall, people with PPPD performed the FSST-VR in a manner similar to controls. However, the opposite relationships identified between self-reported and functional characteristics with head kinematics in people with PPPD vs. controls under “high” visual load suggest that a more detailed investigation of dynamic balance characteristics in people with PPPD is warranted. These results further support the inclusion of spatiotemporal head kinematics as outcomes for dynamic balance tasks, in addition to existing clinical outcomes (e.g. duration). Results from this work will support the development of immersive virtual environments for assessment and treatment of people with PPPD.

## Data Availability

The datasets during and/or analysed during the current study available from the corresponding author on reasonable request.

## Abbreviations

VR: Virtual Reality
VE: Virtual Environment
ABC: Activities-specific Balance Confidence scale
AP ROM: Anteroposterior range of motion
DHI: Dizziness Handicap Inventory
FSST: Four Square Step Test
FSST-VR: Four Square Step Test Virtual Reality
HR: Heart rate
ML ROM: Mediolateral range of motion
TUG: Timed-Up and Go test
STAI-state: State-Trait Anxiety Inventory—state
STAI-trait: State-Trait Anxiety Inventory—trait
SSQ: Simulator Sickness Questionnaire
